# Immunolocalization of a Histidine-Rich Epidermal Differentiation Protein in the Chicken Supports the Hypothesis of an Evolutionary Developmental Link between the Embryonic Subperiderm and Feather Barbs and Barbules

**DOI:** 10.1371/journal.pone.0167789

**Published:** 2016-12-09

**Authors:** Lorenzo Alibardi, Karin Brigit Holthaus, Supawadee Sukseree, Marcela Hermann, Erwin Tschachler, Leopold Eckhart

**Affiliations:** 1 Comparative Histolab and Dipartimento di Scienze Biologiche, Geologiche ed Ambientali (BiGeA), University of Bologna, Bologna, Italy; 2 Research Division of Biology and Pathobiology of the Skin, Department of Dermatology, Medical University of Vienna, Vienna, Austria; 3 Department of Medical Biochemistry, Medical University of Vienna, Vienna, Austria; INSERM, FRANCE

## Abstract

The morphogenesis of feathers is a complex process that depends on a tight spatiotemporal regulation of gene expression and assembly of the protein components of mature feathers. Recent comparative genomics and gene transcription studies have indicated that genes within the epidermal differentiation complex (EDC) encode numerous structural proteins of cornifying skin cells in amniotes including birds. Here, we determined the localization of one of these proteins, termed EDMTFH (Epidermal Differentiation Protein starting with a MTF motif and rich in Histidine), which belongs to a group of EDC-encoded proteins rich in aromatic amino acid residues. We raised an antibody against an EDMTFH-specific epitope and performed immunohistochemical investigations by light microscopy and immunogold labeling by electron microscopy of chicken embryos at days 14–18 of development. EDMTFH was specifically present in the subperiderm, a transient layer of the embryonic epidermis, and in barbs and barbules of feathers. In the latter, it partially localized to bundles of so-called feather beta-keratins (corneous beta-proteins, CBPs). Cells of the embryonic periderm, the epidermis proper, and the feather sheath were immunonegative for EDMTFH. The results of this study indicate that EDMTFH may contribute to the unique mechanical properties of feathers and define EDMTFH as a common marker of the subperiderm and the feather barbules. This expression pattern of EDMTFH resembles that of epidermal differentiation cysteine-rich protein (EDCRP) and feather CBPs and is in accordance with the hypothesis that a major part of the cyclically regenerating feather follicle is topologically, developmentally and evolutionarily related to the embryonic subperiderm.

## Introduction

The cornified skin barrier of amniotes and cornified skin appendages such as claws, hair, and feathers are formed by epidermal keratinocytes that differentiate by inducing the expression of specific sets of genes [1–4]. The stratification of the epidermis begins in the embryo and involves the establishment of the periderm as the superficial layer in all amniotes and the formation of a subperiderm in archosaurs [5–10]. Periderm and subperiderm are shed during late development when a mature cornified layer (stratum corneum) has been established by the definitive epidermis. During adult life the cornified epidermis provides the essential protection against water loss and mechanical stress whereas cornified skin appendages serve various functions including, but not limited to, grasping (claws), thermoinsulation (hair, feathers), and facilitating flight (feathers).

Many of the structural components of cornifying keratinocytes are encoded in a gene cluster termed the Epidermal Differentiation Complex (EDC). In humans and other mammals, the EDC comprises genes for proteins that interact with each other to form, via transglutamination, a cornified cell envelope or with the keratin intermediate filaments during compaction of the cytoskeleton [11, 12]. Recent studies have shown that non-mammalian amniotes also have an EDC in which both orthologs of human EDC genes, such as *loricrin* and *cornulin*, and clade-specific genes which, for example, code for proteins of the scutes of turtle or the feathers of birds are located [13–15]. Proteins traditionally termed beta-keratins [16–18] but now identified as Corneous Beta Proteins (CBPs) represent a major sub-cluster of EDC genes in sauropsids while they are absent in mammals [8, 19]. These proteins of a molecular mass typically in the range of 10–18 kDa possess a characteristic central region (with a most highly conserved stretch of 34 amino acid residues) that folds into an anti-parallel beta-sheet and facilitates the formation of CBP filaments of 3–4 nm thickness [20, 21]. The intra- and intermolecular interactions of sub-domains and sequence motifs in other avian EDC proteins have remained unknown so far. Conserved sequence motifs at the amino- and carboxy-terminus of EDC proteins are likely sites of transglutamination whereas an extremely high cysteine content of epidermal differentation cysteine-rich protein (EDCRP) has been proposed to form multiple disulfide bonds that may contribute to the mechanical strengthening of feathers [22]. Labeling with tritiated histidine and autoradiography suggested that histidine-containing proteins are present in the cytoplasm and in corneous bundles of barbules, however, the identity of these protein(s) was not determined in that study [23].

The morphogenesis and maturation of feathers depends on a complex spatio-temporal cell differentiation program in which EDC-encoded and non-EDC-encoded proteins form the body of the feather whereas other proteins regulate the scaffolding function and programmed cell death of intermediate cells [14, 24–27]. Similarities in the topology and gene expression profiles have suggested that the layered organization of feather follicles is equivalent to that of the embryonic epidermis, with the feather sheath corresponding to the embryonic periderm, the barbules corresponding to the embryonic subperiderm and the marginal plate of barb ridges corresponding to the proliferative layer of the embryonic epidermis proper [7, 9, 28]. As the evolutionary origin of the subperiderm in a common ancestor of birds and alligators [28] predated that of feathers, the evolutionary origin of feathers has probably involved the cooption of embryonic cell type-specification and differentiation mechanisms for a morphogenesis program of a skin appendage that is cyclically renewed in adult birds [22, 28].

One of the EDC proteins of the chicken is EDMTFH (Epidermal Differentiation protein starting with MTF motif, Met-Thr-Phe, and rich in Histidine) [13]. Its expression was detected by RT-PCR in embryonic skin and feathers and, by proteomics, in feathers [13]. Here, we compared the sequence of EDMTFH to that of chicken histidine-rich protein (HRP), also known as fast protein (Fp) [29], a major feather protein [30]. We show that, due to local sequence mismatches, previously reported HRP/Fp sequences are not compatible with the reference genome sequence of the chicken, and we suggest that the amino acid sequence of EDMTFH represents the translation product of the gene that has previously been referred to as HRP or Fp. Furthermore, we demonstrate that EDMTFH is expressed in feather barbules and in the subperiderm, thereby adding support to the hypothesis of a close relationship between these two epithelial derivatives.

## Materials and Methods

### Ethics statement

All animal procedures were approved by the Animal Care and Use Committee of the Medical University of Vienna (Permit Number: 66.016/0014-II/3b/2011), all efforts were made to minimize suffering of animals, and all procedures were conducted according to the guidelines established by the Committee.

### Animals, tissue preparation and fixation

Sexually mature Derco brown (TETRA-SL) laying hens and roosters were purchased from Diglas Co. (Feuersbrunn, Austria), maintained on open floor space with free access to water and feed (standard diet, ssniff, Germany) with a daily light period of 16 hours. For fertilized eggs, hens and roosters were housed together in flocks in the animal facility. Freshly laid and fertilized eggs were incubated at 37.5°C and 60–70% humidity to maintain normal embryonic development. For tissue and organ retrieval, chicken embryos on embryonic days E12 through E19 were euthanized by decapitation.

Tissue samples from chicken embryos were prepared, fixed with 7.5% formaldehyde and embedded in paraffin as described previously [14]. For ultrastructural investigations, skin samples were collected from chick embryos at stages 38–40 as previously reported [31]. The collected tissues were immediately fixed for 5 hours in cold (0–4°C) 4% paraformaldehyde in 0.1 M phosphate buffer at pH 7.4, rinsed in buffer for about 30 minutes, dehydrated in ethanol (70%, 80%, 95%, 100%), and immersed in Bioacryl resin for 3–5 hours (pieces sunk to the bottom of the container) before curing them under ultraviolet light at 0–4°C for 3 days [32].

### Generation of an antibody against EDMTFH

The peptide DHRFKHLYGLHRDHHHD, corresponding to amino acid residues 29–45 of chicken EDMTFH, was synthesized and coupled to keyhole limpet haemocyanin (KLH) by Davids Biotechnologie GmbH, Regensburg, Germany. The KLH-coupled peptide was used as immunogen for the generation of mouse antiserum (Davids Biotechnologie GmbH), essentially according to a published protocol [33]. Immunohistochemistry with antiserum dilutions of 1:250–1:1000 gave specific signals which were not obtained when the primary antibody was omitted or when pre-immune serum or mouse antibodies of unrelated specificities were used instead of anti-EDMTFH.

### Western blot analysis

Chicken embryonic feather samples (stage 38) were homogenized in a solubilization buffer containing 8 M urea, 50 mM Tris-HCl (pH 7.6), 0.1 M 2-mercaptoethanol, 1 mM dithiothreitol and protease inhibitor (Sigma). The particulate material was removed by centrifugation at 10,000 g for 10 minutes. Laemmli buffer was added and samples were denatured at 100°C for 5 minutes. Proteins (40 μg per lane) were separated by sodium dodecyl sulfate-polyacrylamide gel electrophoresis (SDS-PAGE) at a polyacrylamide concentration of 15% using a Biorad apparatus. The Sigma Wide Range molecular weight marker (10–250 kDa) was used for estimating protein masses. After electrophoresis, proteins were transferred onto a nitrocellulose membrane. The membrane was stained with Ponceau Red to visualize the protein transfer. Mouse anti-EDMTFH at a dilution of 1:1000 was used as the primary antibody, and a fluorescence labeled goat anti-mouse immunoglobulin G (IgG-h+I Cy5 conjugated, Bethyl) was used as secondary antibody. Bands were detected using the Biorad external laser Molecular Imager FX combined with the program PharosFX. In negative control experiments, the samples were subjected to the same procedure but the primary antibody was omitted.

### Light and electron microscopy immunolabeling analyses

Immunohistochemical stainings for light microscopy were performed according to a published protocol [14]. Mouse anti-EDMTFH was used at dilutions of 1:250 and 1:500, and biotinylated sheep anti-mouse IgG (1:200; GE, Chalfont, UK) was used as secondary antibody. Sheep serum (10%) was added to the secondary antibody to prevent unspecific binding. Finally, the sections were incubated with streptavidin-biotin-horseradish peroxidase (HRP) complex and 3-amino-9-ethylcarbazole (DakoCytomation, Glostrup, Denmark), and counter-stained with hematoxylin. In control experiments, the primary antibody was either replaced by pre-immune serum or preabsorbed with the antigenic peptide (Davids Biotechnologie GmbH, Regensburg, Germany). The preabsorption procedure was modified from a published protocol [34]. Two μl anti-EDMTFH antibody, 0.5 μl antigenic peptide (10 mg/ml) and 37.5 μl phosphate-buffered saline containing 2% BSA were mixed and incubated at room temperature for 30 min. Subsequently, the antibody was further diluted to the final concentration and used for the immunostaining protocol as described above.

For the electron microscopy study, samples of wing downfeathers (stages 38 Hamburger–Hamilton (HH), n = 3, and 39 HH, n = 3) embedded in Bioacryl resin were sectioned using an ultramicrotome, and 2–4 μm thick sections were collected on glass slides, stained with 1% toluidine blue and observed under a light microscope for general histology. From areas of interest, thin sections of 40–90 nm in thickness were collected on Nickel grids for the immunodetection by immunogold under a transmission electron microscope. In order to improve antibodies penetration, a hatching treatment for 10 minutes with 2% HIO_4_ was done on grid sections, and the grids were rinsed in distilled water for 23 minutes with two changes. Thin sections were pre-incubated for 10 minutes in 0.05 M TRIS-HCl buffer at pH 7.4, containing 1% Cold Water Fish Gelatin. The sections were then incubated for 5 hours at room temperature in primary antibodies diluted 1:100 in buffer. Mouse anti-EDMTFH and, in some experiments, a rabbit “feather keratin” antibody (generously donated from Dr. R. H. Sawyer, University of South Carolina, USA, see [9, 35]) were used as primary antibodies. In controls, the primary antibody was omitted in the first incubation step. The sections were rinsed in buffer and incubated for 1 hour at room temperature with secondary anti-mouse immunoglobulin (IgG) (for detection of EDMTFH) or anti-rabbit IgG (for detection of feather beta keratin) gold-conjugated antibodies (Sigma, USA, 5 or 20 nm gold particles). In double-labeling experiments anti-rabbit IgG 20 nm diameter gold-conjugates and anti-mouse IgG 5 nm diameter gold conjugates were used for the detection of corneous feather beta-proteins (feather keratins) and for the detection of EDMTFH, respectively. After incubation, the grids were rinsed in buffer, dried, stained for 5 minutes with 2% uranyl acetate, and observed under the electron microscope Zeiss 10C/CR operating at 60 kV.

## Results

### EDMTFH corresponds to the previously reported histidine-rich protein (HRP) of chick feather

**Fig 1:**
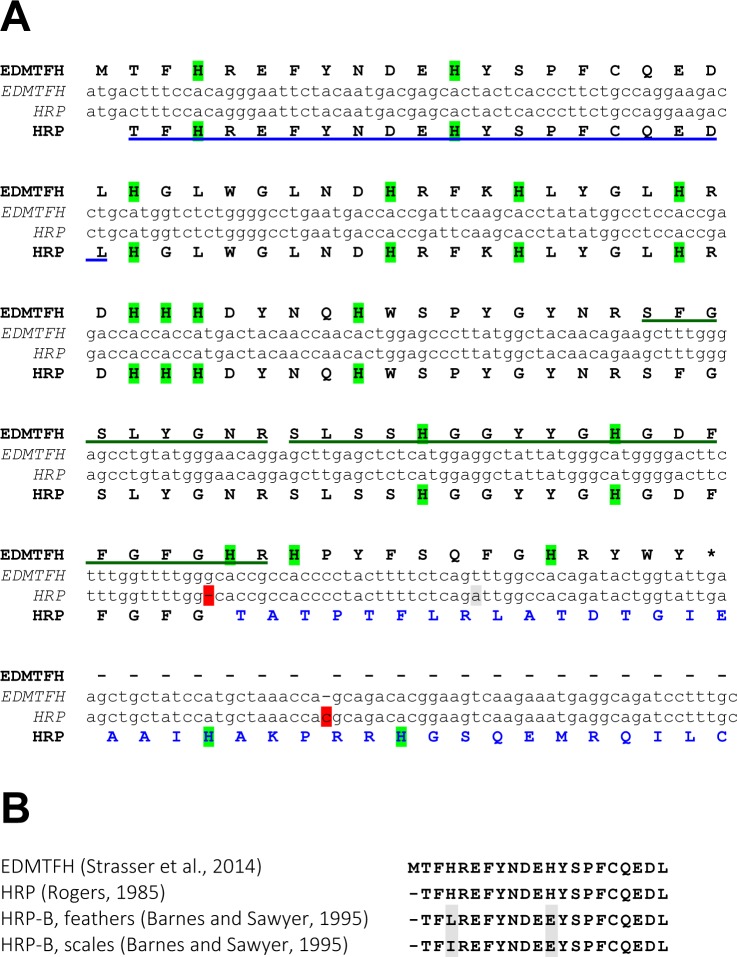
Nucleotide and amino acid sequence alignments of EDMTFH versus histidine-rich protein (HRP). (**A**) The nucleotide sequences of the coding region of chicken EDMTFH [13] and of the chicken HRP cDNA reported previously [29] were aligned. Translations into amino acid sequences are shown above and below the sequences, respectively. Note that insertions and deletions (red shading) in the cDNA sequence relative to the chicken EDMTFH gene in the current genome assembly cause reading frameshifts leading to the prediction of a different carboxy-terminus of HRP (blue fonts) relative to EDMTFH. Sequences corresponding to peptides that were previously identified in feather extracts are marked underlined feather proteins peptides (underlined) corresponding to EDMTFH were identified by (blue underline [29], green underlines [13]). Histidine (H) residues are highlighted by green shading. The stop codon of EDMTFH is marked with an asterisk. (**B**) Alignment of amino-terminal amino acid sequences of EDMTFH [13] and HRP, as determined by direct sequencing of proteins isolated from feathers [29, 38]. Predicted HRP-B residues that deviate from the EDMTFH sequence at positions of histidines (H) are shaded grey.

Amino acid sequence alignments showed that EDMTFH is identical to the previously reported chicken histidine-rich protein (HRP) [29, 36] with the exception of the carboxy-terminal segment (Fig 1). Re-investigation of the previously published cDNA sequence from which the carboxy-terminus of HRP had been derived by translation *in silico* [29] suggested that two indel changes in the nucleotide sequence, inducing a frameshift relative to the chicken genome sequence and the sequence of EDMTFH cDNA [13], had caused an incorrect prediction of the carboxy-terminal amino acid sequence of HRP (Fig 1A). Our previous search for EDMTFH peptides in the chicken feather proteome [13, 37] revealed two EDMTFH-derived peptides (Fig 1A, green underlines) of which one comprised a part of the carboxy-terminal amino acid sequence present in EDMTFH but not in the predicted HRP.

The amino-terminal sequence of EDMTFH is identical to a 20-amino acid peptide previously identified by direct peptide sequencing of HRP [29] (Fig 1A, blue underline) and highly similar to the sequences of peptides reported for so-called HRP-B proteins [38] (Fig 1B). The 5´-untranslated region of HRP/Fp [39] matches perfectly to the non-coding sequences in exon 1 and at the 5´-end of exon 2 of *EDMTFH* (S1 Fig), while the coding sequence of the HRP cDNA [29] (with the sequence differences shown in Fig 1A) is entirely derived from exon 2 of the *EDMTFH* gene (S1 Fig). As the EDMTFH sequence, determined from a chicken cDNA [13], matches perfectly with the chicken reference genome sequence whereas the previously reported HRP and HRP-B sequences show only partial identities, we keep using the name EDMTFH instead of HRP.

### EDMTFH belongs to a group of epidermal differentiation proteins rich in aromatic amino acid residues

**Fig 2:**
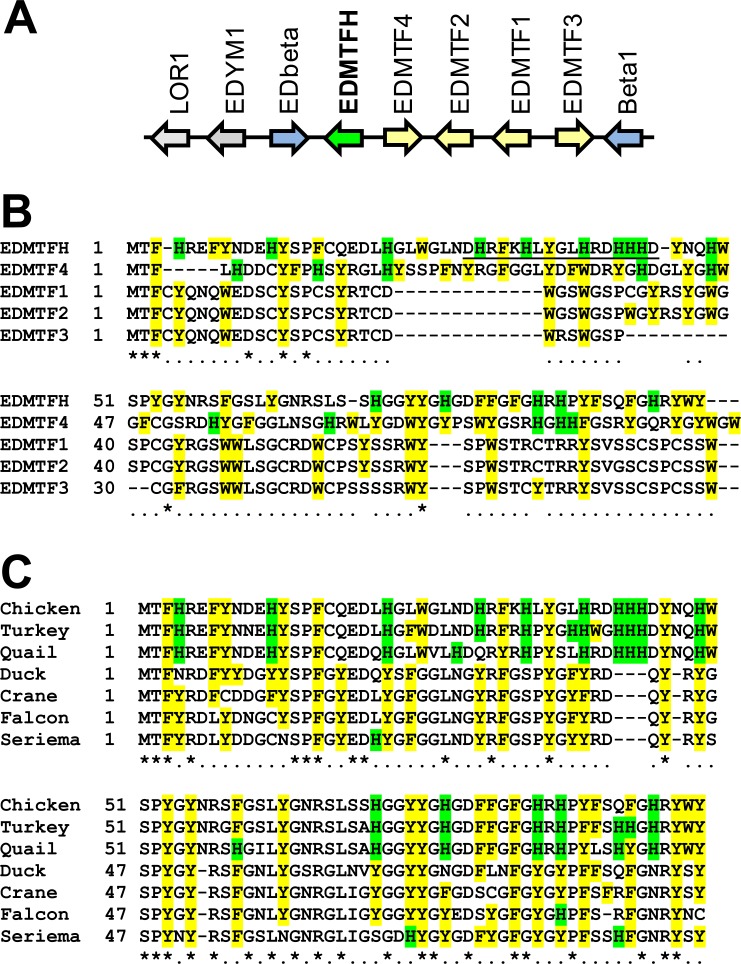
Comparison of chicken EDMTFH versus other EDMTF proteins of the chicken and homologs in other species. (**A**) EDMTFH gene locus in the chicken. Arrows indicate the origientation of gene transcription. The genes *EDbeta* and *Beta1* (preliminary names [13]) encode corneous beta-proteins (CBPs), also known as beta-keratins. (**B**) Amino acid sequence alignment of EDMTFH versus other chicken EDMTF proteins. The peptide used as an epitope for raising the anti-EDMTFH antibody is underlined. (**C**) Amino acid sequence alignment of chicken EDMTFH versus the most similar proteins of other birds. Formats in (**B**) and (**C**) indicate the following: Histidine (H) residues are highlighted by green shading, other aromatic residues are highlighted by yellow shading. Numbers indicate amino acid sequence positions. Identity of residues in all sequences is indicated by an asterisk and conservation in at least 50% of the sequences is indicated by "." below the alignments. Hyphens were introduced to maximize the alignment of the sequences. Sequences of EDMTFH orthologs of turkey (*Meleagris gallopavo*) and quail (*Coturnix japonica*) were predicted from genomic DNA. Accession numbers of other EDMTFH sequences: AHA62422.1 (chicken, *Gallus gallus*), XP_012964640.1 (duck, *Anas platyrhynchos*), XP_010299618.1 (crane, *Balearica regulorum gibbericeps*), XP_013153676.1 (falcon, *Falco peregrinus*), XP_009701209.1 (seriema, *Cariama cristata*).

The EDMTFH gene is located in the EDC and is flanked by the CBP gene EDbeta and EDMTF4 [13] (Fig 2A). EDMTF4 is most similar to EDMTFH among chicken proteins, followed by EDMTF1 through 3, which are located next to EDMTF4 (Fig 2B). An internal peptide of EDMTFH that differs in sequence from all its EDMTF paralogs (Fig 2B, underlined) was selected as an immunogen for raising an EDMTFH-specific antibody for *in situ* immunolocalization studies (see below). The cysteine contents of EDMTFH and EDMTF4 are much lower than that of other EDMTF proteins (1–2% versus 11–13%). A high histidine content is present only in EDMTFH, however, aromatic amino acids (F, W, Y, and H) are enriched in all EDMTF proteins.

To determine the evolutionary conservation of EDMTFH among birds, we screened avian genome and protein data using the amino acid sequence of chicken EDMTFH as a query. Homologs of EDMTFH were identified in all birds investigated and the carboxy-terminal sequence was highly conserved (Fig 2C). The genes encoding EDMTFH homologs in other species were located at genome positions of shared synteny as compared to chicken EDMTFH and the encoded proteins showed higher sequence similarity to EDMTFH than to other chicken proteins.

Histidine was present at high amounts in EDMTFH of chicken, turkey and quail, which are representatives of the family Phasianidae (Fig 2C). In other birds, the proteins most similar to EDMTFH had lower contents of histidine (15.2% in chicken EDMTFH) but shared with EDMTFH the high content of aromatic residues (40.4% in chicken EDMTFH). Notably, chicken EDMTFH (total number of amino acid residues: 99) contains 12 sites in which an aromatic residue is followed by glycine, and a similar enrichment for such dipeptides is present in the most similar proteins of other avian species (Fig 2C). Together, the sequence features of EDMTFH and their differential conservation during evolution suggest that the high histidine content arose specifically in the avian clade Phasianidae while the high content of aromatic amino acid residues is conserved and therefore likely important for the function of EDMTFH.

### EDMTFH is present in the subperiderm and in feather barbules

**Fig 3:**
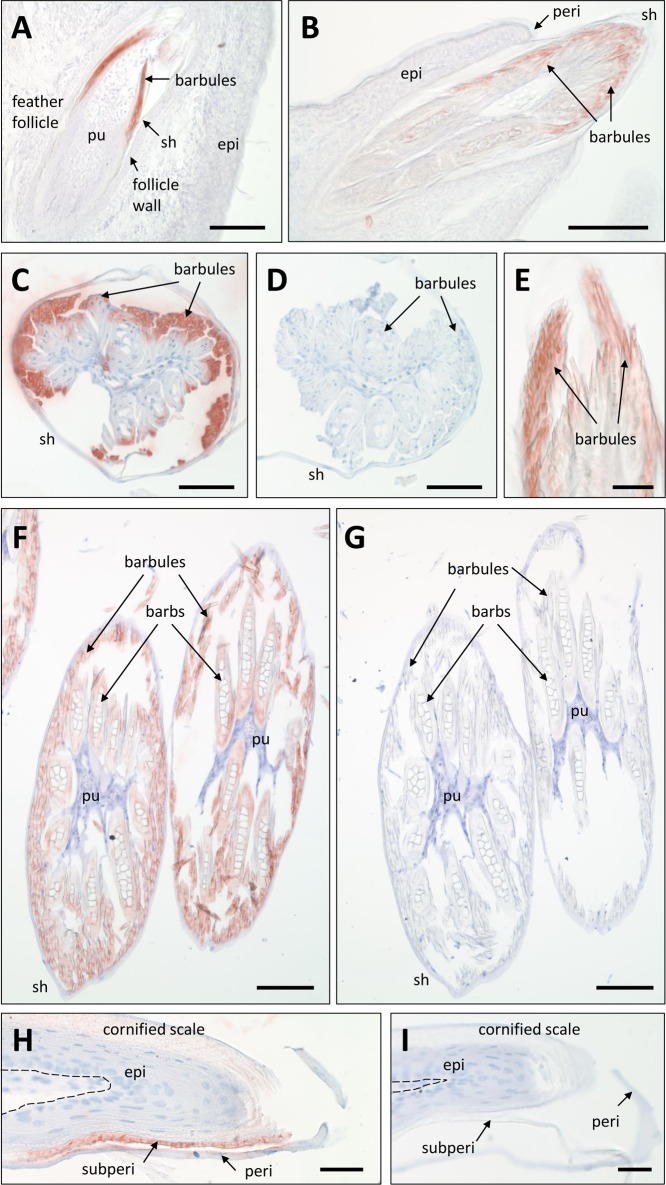
Light microscopic immunohistochemistry of EDMTFH. (**A-C**, **E, F**) Feather follicles and feathers on the wings of chick embryos on day E18 of development were immunostained with anti-EDMTFH (red). In negative control experiments the primary antibody was replaced with an antiserum raised against an unrelated peptide (**D**) or preabsorbed with the antigen (**G**). Scutate scales on the legs of chick embryos (day E19) were immunolabeled for EDMTFH (**H**) or subjected to the negative control experiment (**I**). The dermo-epidermal junction is indicated by a dashed line (**H**, **I**). epi, epidermis; peri, periderm; pu, pulp; sh, feather sheath; subperi, subperiderm. Bars: 100 μm (**A**, **B**, **F**, **G**), 50 μm (**C**-**E**), 25 μm (**H**, **I**).

For *in situ* immunolocalization studies, antibodies were raised against the EDMTFH-specific peptide DHRFKHLYGLHRDHHHD (Fig 2B). Western blot analysis confirmed that the antibody bound to a feather protein of the expected size of approximately 12 kDa whereas larger proteins such as CBPs and keratins were not labeled (S2 Fig). Embryonic skin and feather follicles as well as adult skin of chickens were immunohistochemically stained with this antiserum and, as negative controls, with preimmune serum and antibodies of unrelated specificities. Furthermore, in some control experiments the primary antibody was preadsorbed with the antigenic peptides. EDMTFH was most strongly expressed in growing feathers on embryonic days E14 and E18 whereas the skin between feather follicles was EDMTFH-negative. EDMTFH was concentrated in barbule cells (Fig 3A–3C, 3E and 3F) whith external cells of barbule plates being most strongly labeled (Fig 3C). Barb cortical and medullary cells were also labeled for EDMTFH (Fig 3F), however, barbs (rami) were immunonegative in feathers that appeared to have progressed further in differentiation, perhaps indicating that the EDMTFH epitope was masked during cornification. Negative control experiments did not yield staining of barbules and, thereby, confirmed the specificity of the EDMTFH immunostaining (Fig 3D and 3F). The feather sheath lacked EDMTFH (Fig 3A–3C and 3F).

EDMTFH was also expressed in the subperiderm of scutate scales on the legs of chicks on embryonic days E18 and E19 (Fig 3H). The immunolabeling pattern was irregular, and the labeling intensity appeared to decrease with cornification of the subperidermal cells. Negative control stainings in which the primary antibody was either replaced by the preimmune serum or preabsorbed with the antigen were negative, confirming the specificity of the staining (Fig 3I). Other parts of the embryonic skin, including the dermis, the basal and lower suprabasal layers of the epidermis, the cornified cell layers of scales and the periderm consistently lacked expression of EDMTFH (Fig 3A).

### Ultrastructural localization of EDMTFH

**Fig 4:**
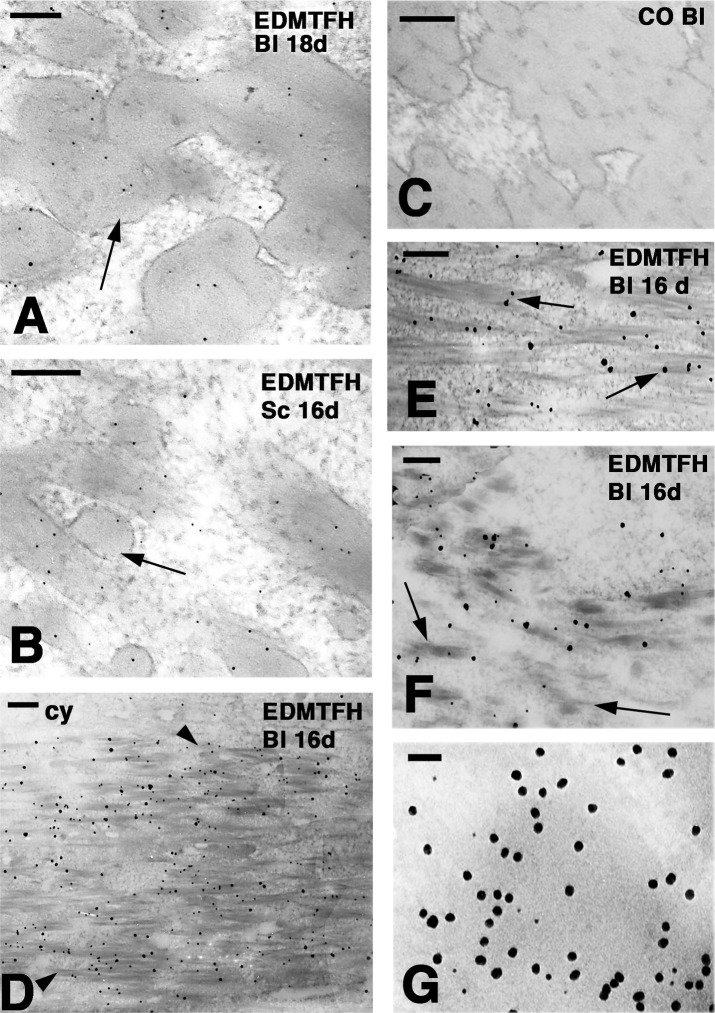
Ultrastructural localization of EDMTFH by immunogold labeling. Downfeathers and scales of chicken embroys at days 16 (16d) and 18 (18d) of development were labeled for EDMTFH either without (**A-C, G**) and with (**D-F**) silver enhancement. (**A**) Diffuse labeling over corneous bundles (arrow) of barbule cells (bl). (**B**) Diffuse labeling in the corneous bundles (arrow) of a subperiderm cell in a scale. (**C**) Immuno-negative control section of a barbule. (**D**) Labeling cytoplasmic corneous bundles (arrowheads) but not the cytoplasm (cy) in a barbule cell (bl). (**E**) Close-up to show the association of the labeling with corneous bundles (arrows). (**F**) Early differentiating barbule cell with short corneous bundles (arrows). (**G**) Double-labeling for EDMTFH (5 nm gold particles) and feather beta-keratin (20 nm gold particles) in a barbule cell. Note that the large particles appear to be more abundant than the small particles. A lower magnification image of the double-labeling is shown in S3 Fig. Bars: 100 nm (**A**, **B**); 200 nm (**C-F**); 50 nm (**G**).

To determine the subcellular localization of EDMTFH, immunogold labeling and transmission electron microsopy were performed using the anti-EDMTFH antibody. EDMTFH immunogold labeling was consistently detected in the external barbules cells while it became uneven in barb cortical cells and disappeared in barb medullary cells of the feather samples investigated here. The labeling was mainly, but not exclusively, observed over the dense CBP packets accumulated among the paler cytoplasm (Fig 4A, S3 Fig). A similar diffuse labeling was detected in the subperiderm of scales (Fig 4B). No antibody-conjugated gold particles were present in control sections (Fig 4C). The gold labeling with the anti-EDMTFH antibody over the linear filaments of most barbule cells and some barb cortical cells was further observed after silver enhancement, a technique that increases the size of the ultrastructural label to allow a more panoramic view of the labeling over broader areas of barbule cells (Fig 4D–4F). Double labeling with anti-EDMTFH, conjugated to 5 nm gold particles, and anti-feather CBP, conjugated to 20 nm gold particles, suggested that EDMTFH at least partly co-localized with feather CBP (Fig 4G). The labeling for feather CBP was observed over the corneous bundles in most barbule and barb cells and, consequently, the entire cell appeared labeled, except for the central cytoplasm where scattered gold particles were present. The total amount of label for EDMTFH appeared to be lower than that for feather CBP, and in contrast to the even distribution of feather CBP, EDMTFH was variably concentrated in different areas of barbule cells (S3 Fig).

## Discussion

**Fig 5:**
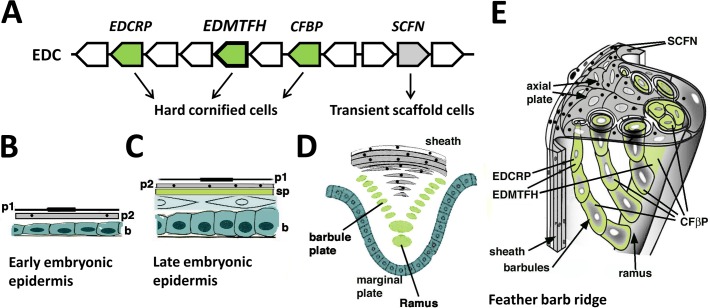
Schematic representation of the contribution of EDMTFH to feather cornification. (**A**) EDMTFH and other genes, that encode proteins of the feather follicle, are located in the same gene cluster, the avian epidermal differentiation complex (EDC). EDMTFH, EDCRP and corneous feather beta proteins (CFBPs) are components of hard cornified cells whereas scaffoldin (SCFN) is a component of cells that form a transient scaffold of growing feathers. (**B-D**) The embryonic epidermis of birds increases the number of layers during development. During late development, 2 layers of periderm (p1, p2) and a subperiderm (sp) are present above the definitive epidermis (**C**). The feather barb ridge has a topologically similar organization as late embryonic epidermis on scutate scales whereby the equivalents of the periderm form the feather sheath and the axial plate, and barbules cells and rami are equivalent to the subperiderm (**D**), as suggested by common expression of protein markers. A 3-dimensional depiction of a growing barb ridge of a down feather indicates the different roles of cornifying cells (green) and transient scaffolding cells (grey) in the morphogenesis of feathers (**E**). Periderm granules containing scaffoldin are indicated as black dots.

The results of this study show that EDMTFH is expressed in the subperiderm of the embryonic epidermis and in barbs and barbules of feathers (Fig 5). Our data improve and extend previous studies in which HRP/Fp was suggested to have a main role in feathers [29, 30, 36]. Together with comparative sequence analysis, the immunolabeling results point to a role of EDMTFH in the maturation of the cornified components of mature feathers.

Research on EDMTFH, then termed HRP or Fp, was initiated in the 1970ies and yielded the important insight that a histidine-rich protein is present at relatively high amounts in chicken feathers as well as in embryonic epidermis [29, 30, 36, 38, 39]. Revisiting this topic, we found that a published cDNA sequence of chicken HRP contained nucleotide changes relative to the chicken genome sequence, leading to an aberrant prediction of the carboxy-terminus of the protein [29]. In our studies, an EDMTFH cDNA (GenBank accession number KC963987) [13] showed sequence identity to the chicken genome sequence, and a peptide identified by proteomic analysis of feathers supported the carboxy-terminal sequence of EDMTFH but not that of HRP (Fig 1A). These data validate the EDMTFH sequence presented here and indicate that the prediction of the carboxy-terminus of the HRP sequence was caused by the presence of a polymorphism in the animal from which the HRP mRNA was isolated [29] or, more likely, by a cDNA cloning artifact.

The antibody that was generated in our study was directed to an internal EDMTFH-specific peptide. Detection by Western blot and immunochemical staining of EDMTFH in chicken feathers confirmed peptide isolation and sequencing [29] and proteomics results of feathers (see [13]). The generation of the EDMTFH antibody facilitated the localization of this protein *in situ*. Immunohistochemical staining could be done by standard antigen retrieval with citrate buffer (pH 6) whereas ultrastructural immunogold labeling required an etching protocol [31]), indicating that the EDMTFH epitope was at least partly masked in the embedded tissues. Labeling was observed in many cells of feather barbules and in the subperiderm of scutate scales, however, it is possible that the epitope of EDMTFH is masked by tight protein-protein interactions including transglutamination so that a fraction of the EDMTFH proteins is not accessible for immunolabeling. In agreement with this notion, EDMTFH immunolabeling was detected in the rami (barbs) of many but not all developing feathers. Thus, the immunolabeling pattern likely represents the distribution of EDMTFH at cellular terminal differentiation stages prior to full cornification.

The present study shows that EDMTFH is expressed in feather cells that undergo hard cornification, i.e. the conversion into components of a hard cornified skin appendage [40, 3] (Fig 5). Double-labeling of EDMTFH protein and feather CBPs at the ultrastructural level (Fig 4G) supported the expectation that in these cells EDMTFH is less abundant than CBPs. The latter are encoded by a family of more than a hundred genes, many of which are co-expressed in all types of feathers [25]. The EDMTFH protein appeared to be added to the corneus bundles of barbule cells while it was present at smaller (immuno-detectable) amounts in barb cortical and absent in medullary cells. These results indicate that EDMTFH is one of the structural proteins, besides CBPs, that form the cytoskeleton of maturing feathers.

The expression pattern of EDMTFH is similar to that of EDCRP and clearly different from that of scaffoldin, another EDC-encoded protein that we have previously detected in the embryonic periderm and feather sheath [14, 41]. EDCRP has an extraordinarily high content of cysteine residues and, therefore, it likely forms multiple disulfide bonds which might contribute to the cross-linking of cytoskeletal proteins and the hardening of feathers. This process is supposed to resemble the maturation of mammalian hair fibers in which cysteine-rich keratin-associated proteins (KAPs, also known as Krtaps) are expressed [22, 42]. Interestingly, another class of KAPs is rich in glycine and tyrosine [43], and these proteins have been suggested to contribute to the mechanical properties of hair by establishing bidirectional protein interactions via cation-π interactions or π stacking [44, 45]. The latter type of protein-protein interactions depends on regularly arranged aromatic residues (in this case, tyrosine). It is interesting to note that glycine and tyrosine-rich KAPs are similar to EDMTF proteins, including EDMTFH, with regard to size and amino acid sequence (S4 Fig). As both types of proteins are expressed at sites of hard cornification (KAPs in hair, EDMTFH in feathers), functional analogy may be presumed. This hypothesis remains to be tested in future studies.

EDMTFH and related EDMTF proteins are conserved among diverse species of birds whereby only the high content of aromatic residues but not the high content of histidine is conserved outside the clade comprising chicken, turkey and quail (Phasianidae) (Fig 2C). EDMTFH is not homologous to mammalian filaggrin, which has also been referred to as histidine-rich protein [46]. Filaggrin is an S100 fused-type protein encoded by a gene in the mammalian EDC [47, 12]. It has a histidine content of 10%, undergoes proteolytic degradation and gives rise to free histidine as a precursor of the UV-absorbing substance urocanic acid in the cornified layer of the epidermis of mammals, whereas it is absent in birds [48–50, 14]. A recent paper found that a chromosomal locus containing *EDMTFH* appeared to be associated with red feather coloration in a crossing experiment of common canaries and red siskins [51]. However, no mechanistic link between *EDMTFH* and the color of feathers was identified. In further studies, it will be interesting to investigate whether adaptation of EDMTF genes, such as the rise of the histidine content of the EDMTFH protein in Phasianidae, was associated with specific changes in feather properties.

Our results demonstrate that, besides the feather follicle, EDMTFH is expressed in the subperiderm of the embryonic epidermis (Figs 3–5). The immunolabeling obtained with the anti-EDMTFH antibody is similar to the previously reported distribution pattern of the so-called HRP-B protein, as determined using an antibody against HRP isolated from feathers [38]. This congruence of immunolabelings further supports the identity of EDMTFH and HRP, provided that the previously reported HRP sequence is corrected at the carboxy-terminus as outlined in Fig 1). A similar expression pattern as that of EDMTFH/HRP has been detected, by immunohistochemistry, for feather-type CBP (beta-keratin) [9, 35], and, by mRNA *in situ* hybridization, for EDCRP [22]. Together, these studies provide substantial amount of evidence in support of the hypothesis that feather barbs and barbules are related, in terms of evolution and development, to the embryonic subperiderm [7, 9, 22, 29, 52]. Further studies of EDMTFH may help to shed more light into the molecular basis of the evolutionary origin, the growth and the material properties of feathers.

## Supporting Information

S1 Fig The nucleotide sequence of a partial HRP/Fp cDNA matches the 5´-untranslated region of EDMTFH. (**A**) Nucleotide sequence published by Presland and colleagues [39]. (**B**) Alignment of the HRP/Fp cDNA sequence and the *EDMTFH* gene sequence. The *EDMTFH* sequence was derived from the current chicken reference genome sequence (GenBank Accession number NC_006112.3). Nucleotide number 1 of *EDMTFH* in this alignment corresponds to NC_006112.3 nucleotide number 1977520, and *EDMTFH* is transcribed from the minus strand. The proximal promoter region including a TATA box-like element (underlined) is shown with blue fonts. Intronic sequences are marked by red fonts. The start codon is highlighted by green shading. (**C**) Schematic depiction of the exon-intron structure of the chicken *EDMTFH* gene. Color code: blue, non-transcribed regions flanking the gene; black, exons; red, intron; green, start codon; yellow, stop codon.
(PDF)

S2 Fig Western blot analysis using the anti-EDMTFH antibody. Protein was extracted from embryonic feathers of chicken, electrophoresed through a 15% polyacrylamide gel and blotted onto a nitrocellulose membrane. After Ponceau staining of total protein (right panel), the membrane was probed with anti-EDMTFH (primary antibody) and fluorescence-labeled goat anti-mouse immunoglobulin G (secondary antibody). In the negative (neg.) control experiment, the primary antibody was omitted. Positions of molecular mass markers are indicated on the left. kDa, kilo-Dalton.
(PDF)

S3 Fig Double immunolabeling of EDMTFH and feather-type corneous beta protein in barbule cells. Low magnification view of double (DOUB) immunolabeling for EDMTFH (small gold particles, highlighted by red circles) and feather corneous beta protein (large gold particles) in barbule cells at stage 37–38 of development. Feather beta keratin labeling was concentrated over beta packets (dark) that are surrounded by the less electron-dense cytoplasm (cy). EDMTFH labeling is sparse in both cytoplasm and beta packets. n, nucleus. Bar, 200 nm.
(PDF)

S4 Fig Amino acid sequence alignment of chicken (*Gallus gallus*, Gg) EDMTFH and human (*Homo sapiens*, Hs) keratin-associated protein (KAP/Krtap)7-1. Positions of identical residues in both proteins are indicated by * below the alignment. Conservation of aliphatic (I, L, M, V) and hydrophilic (S, T, N, Q, E, D, K, R) residues in indicated by ":" and ".". Aromatic residues (F, H, W, Y) are highlighted by yellow shading and glycine (G) residues are highlighted by grey shading.
(PDF)
