# Supplementary material for: Immunolocalization of a Histidine-Rich Epidermal Differentiation Protein in the Chicken Supports the Hypothesis of an Evolutionary Developmental Link between the Embryonic Subperiderm and Feather Barbs and Barbules
Source: PLoS One. 2016 Dec 9;11(12):e0167789. doi: 10.1371/journal.pone.0167789 (PMC5147990; doi:10.1371/journal.pone.0167789)

A

```
>HRP/Fp_partial (Presland et al. 1989)
attcgcttctctcgagttcctctcctcggtgaaccgggtttccctccaacaaccagcaatg
```

B

HRP/Fp cDNA (Presland et al., 1989) vs. *EDMTFH* gene (Gallus gallus 5.0)

Exon 1:

```
EDMTFH   1 ttgagagggtatatatacagacctcgagctccggagcttcattcgcttctctcgagttcc
HRP/Fp   1 ----------------------------------------attcgcttctctcgagttcc

EDMTFH  61 tctcctcggtgaaccgggtaagtccacactgcctatactctactcttcttgatatgtctt
HRP/Fp  21 tctcctcggtgaaccgg-------------------------------------------
```

Exon 2:

```
EDMTFH 771 gttccctgctgtgtgtttcaggtttccctccaacaaccagcaatgactttccacagggaa
HRP/Fp  38 ---------------------gtttccctccaacaaccagcaatg---------------
```

C

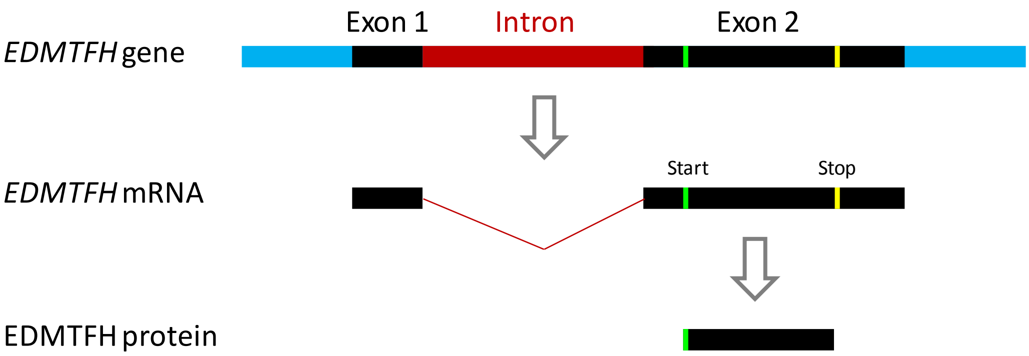

Supplement: S1 Fig — (A) Nucleotide sequence published by Presland and colleagues [39]. (B) Alignment of the HRP/Fp cDNA sequence and the EDMTFH gene sequence. The EDMTFH sequence was derived from the current chicken reference genome sequence (GenBank Accession number NC_006112.3). Nucleotide number 1 of EDMTFH in this alignment corresponds to NC_006112.3 nucleotide number 1977520, and EDMTFH is transcribed from the minus strand. The proximal promoter region including a TATA box-like element (underlined) is shown with blue fonts. Intronic sequences are marked by red fonts. The start codon is highlighted by green shading. (C) Schematic depiction of the exon-intron structure of the chicken EDMTFH gene. Color code: blue, non-transcribed regions flanking the gene; black, exons; red, intron; green, start codon; yellow, stop codon. (PDF) [file pone.0167789.s001.pdf]
