# Supplementary figures and images for: Immunolocalization of a Histidine-Rich Epidermal Differentiation Protein in the Chicken Supports the Hypothesis of an Evolutionary Developmental Link between the Embryonic Subperiderm and Feather Barbs and Barbules
Source: PLoS One. 2016 Dec 9;11(12):e0167789. doi: 10.1371/journal.pone.0167789 (PMC5147990; doi:10.1371/journal.pone.0167789)

kDa

250

150

100

75

50

37

25

20

15

10

anti-EDMTFH

neg. control

Ponceau

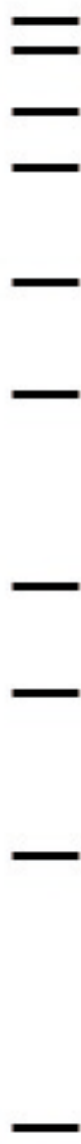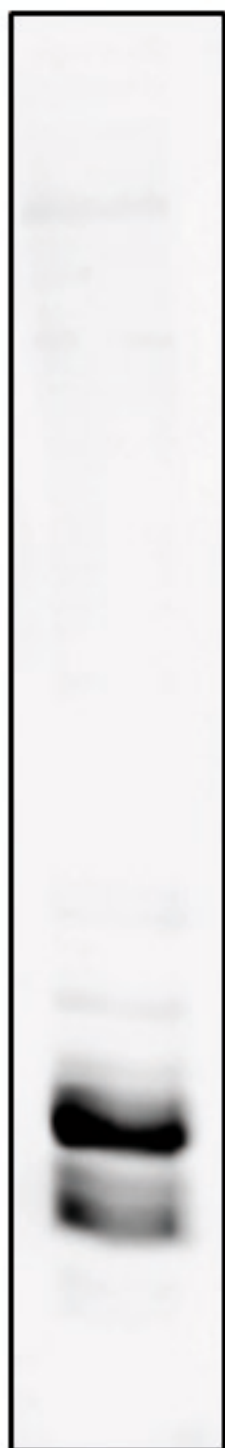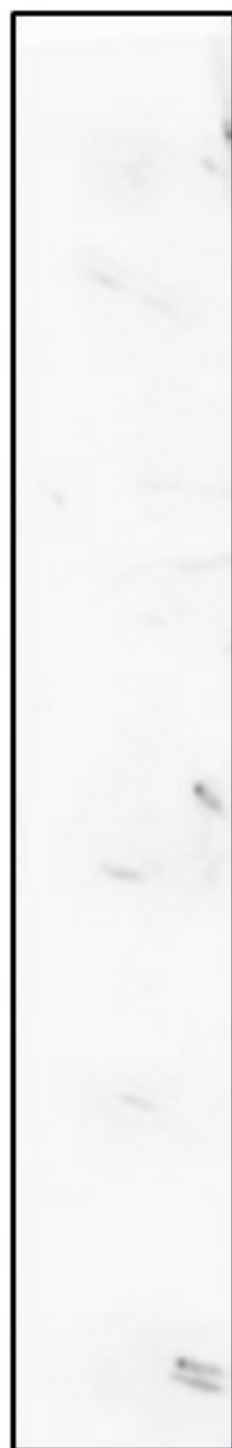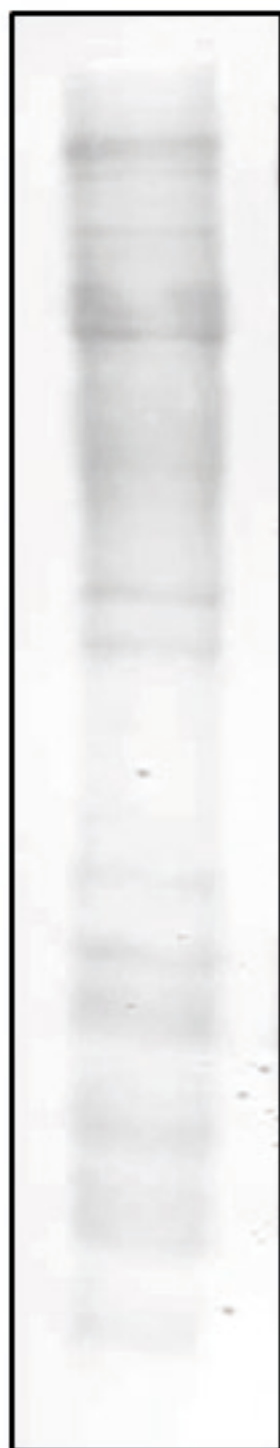

Supplement: S2 Fig — Protein was extracted from embryonic feathers of chicken, electrophoresed through a 15% polyacrylamide gel and blotted onto a nitrocellulose membrane. After Ponceau staining of total protein (right panel), the membrane was probed with anti-EDMTFH (primary antibody) and fluorescence-labeled goat anti-mouse immunoglobulin G (secondary antibody). In the negative (neg.) control experiment, the primary antibody was omitted. Positions of molecular mass markers are indicated on the left. kDa, kilo-Dalton. (PDF) [file pone.0167789.s002.pdf]

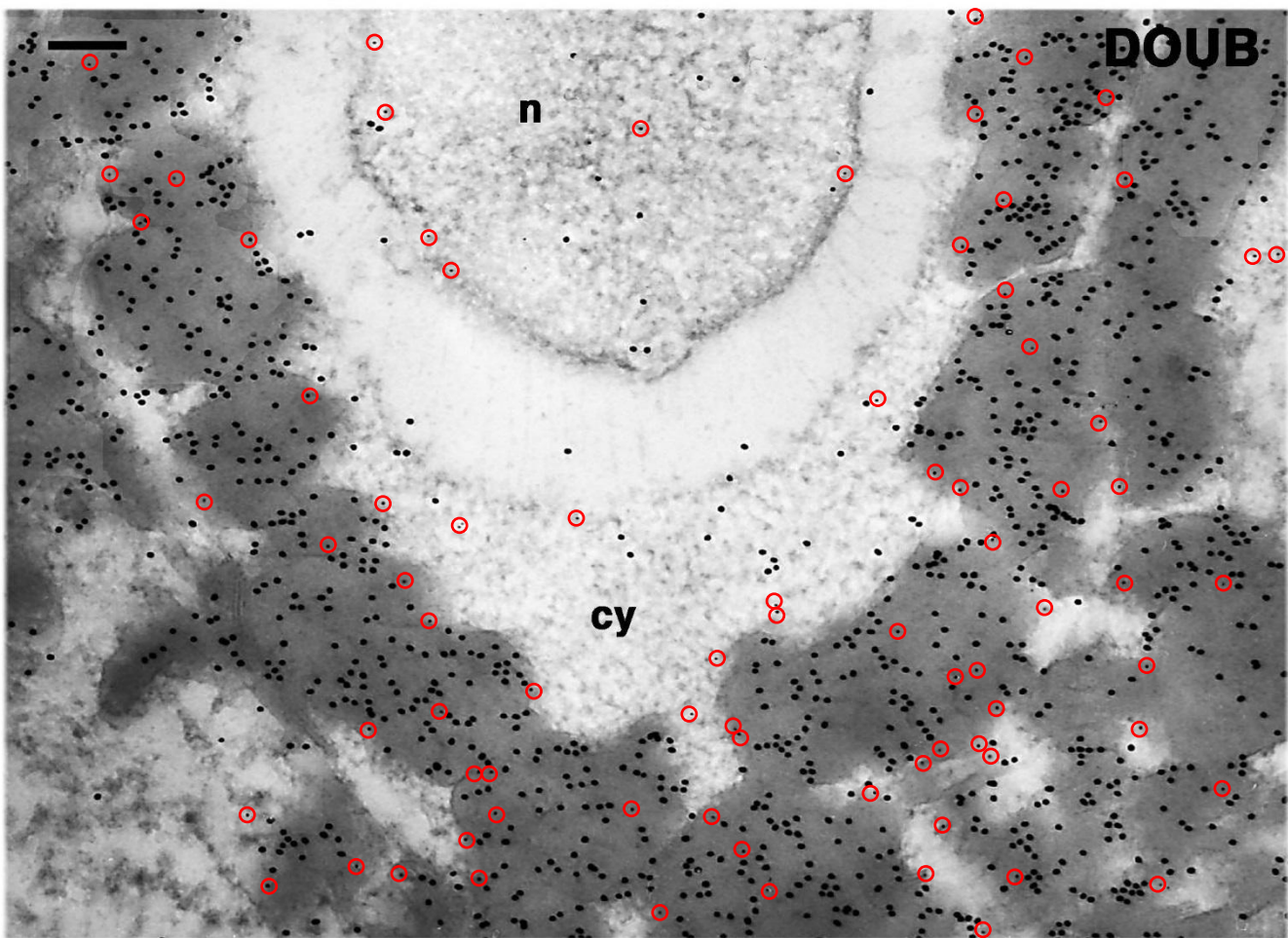

Supplement: S3 Fig — Low magnification view of double (DOUB) immunolabeling for EDMTFH (small gold particles, highlighted by red circles) and feather corneous beta protein (large gold particles) in barbule cells at stage 37–38 of development. Feather beta keratin labeling was concentrated over beta packets (dark) that are surrounded by the less electron-dense cytoplasm (cy). EDMTFH labeling is sparse in both cytoplasm and beta packets. n, nucleus. Bar, 200 nm. (PDF) [file pone.0167789.s003.pdf]
