# Supplementary material for: Immunolocalization of a Histidine-Rich Epidermal Differentiation Protein in the Chicken Supports the Hypothesis of an Evolutionary Developmental Link between the Embryonic Subperiderm and Feather Barbs and Barbules
Source: PLoS One. 2016 Dec 9;11(12):e0167789. doi: 10.1371/journal.pone.0167789 (PMC5147990; doi:10.1371/journal.pone.0167789)
Supplement: S4 Fig — Positions of identical residues in both proteins are indicated by * below the alignment. Conservation of aliphatic (I, L, M, V) and hydrophilic (S, T, N, Q, E, D, K, R) residues in indicated by ":" and ".". Aromatic residues (F, H, W, Y) are highlighted by yellow shading and glycine (G) residues are highlighted by grey shading. (PDF) [file pone.0167789.s004.pdf]

A

```
Gg_EDMTFH  1  MTFHREFYNDEHYSPFCQEDLHGLWGLNDHRFKHLYGLHRDHHHDYNQHW
Hs_KAP7-1  1  MTRYFCCGSYFPGYPIYGTNFHGTFRATPLNCVVPLGSPLNYGCGCN---
              ** :    .     *   .: ** :  .  .     *   ::    *

Gg_EDMTFH  51 SPYGYNRSFGSLYGNRSLSSHGGYYGHGDFFGFGHRHPYFSQFGHRYWY
Hs_KAP7-1  48 ---GYS-SLGYSFGGSNINNLGGCYGGSFYRPWGSG----SGFGYST-Y
                 **. * *  :* ..:.. ** **   :  :*      * **:.  *
```

B

Gg_EDMTFH

Number of amino acids: 99
Molecular weight: 12175.1
Theoretical pI: 6.94
Amino acid composition:

Ala	(A)	0	0.0%
Arg	(R)	7	7.1%
Asn	(N)	5	5.1%
Asp	(D)	6	6.1%
Cys	(C)	1	1.0%
Gln	(Q)	3	3.0%
Glu	(E)	3	3.0%
Gly	(G)	13	13.1%
His	(H)	15	15.2%
Ile	(I)	0	0.0%
Leu	(L)	7	7.1%
Lys	(K)	1	1.0%
Met	(M)	1	1.0%
Phe	(F)	10	10.1%
Pro	(P)	3	3.0%
Ser	(S)	8	8.1%
Thr	(T)	1	1.0%
Trp	(W)	3	3.0%
Tyr	(Y)	12	12.1%
Val	(V)	0	0.0%

Aromatic 40.4%

C

Hs_KAP7-1

Number of amino acids: 87
Molecular weight: 9288.3
Theoretical pI: 8.57
Amino acid composition:

Ala	(A)	1	1.1%
Arg	(R)	3	3.4%
Asn	(N)	7	8.0%
Asp	(D)	0	0.0%
Cys	(C)	6	6.9%
Gln	(Q)	0	0.0%
Glu	(E)	0	0.0%
Gly	(G)	19	21.8%
His	(H)	1	1.1%
Ile	(I)	2	2.3%
Leu	(L)	5	5.7%
Lys	(K)	0	0.0%
Met	(M)	1	1.1%
Phe	(F)	7	8.0%
Pro	(P)	6	6.9%
Ser	(S)	10	11.5%
Thr	(T)	5	5.7%
Trp	(W)	1	1.1%
Tyr	(Y)	11	12.6%
Val	(V)	2	2.3%

Aromatic 22.8%
